# Cold Treatment Breaks Dormancy but Jeopardizes Flower Quality in *Camellia japonica* L.

**DOI:** 10.3389/fpls.2015.00983

**Published:** 2015-11-12

**Authors:** Andrea Berruti, Annelies Christiaens, Ellen De Keyser, Marie-Christine Van Labeke, Valentina Scariot

**Affiliations:** ^1^Institute for Sustainable Plant Protection, National Research CouncilTorino, Italy; ^2^Department of Agricultural, Forest and Food Sciences, University of TorinoGrugliasco, Italy; ^3^Department of Plant Production, Faculty of Bioscience Engineering, Ghent UniversityGhent, Belgium; ^4^Plant Sciences Unit, Institute for Agricultural and Fisheries ResearchMelle, Belgium

**Keywords:** abscisic acid, RT-qPCR, dormancy breaking, chilling requirement, anthocyanin

## Abstract

*Camellia japonica* L. is an evergreen shrub whose cultivars are of great ornamental value. In autumn, after flower bud differentiation, dormancy is initiated. As in many other spring flowering woody ornamentals, winter low temperatures promote dormancy release of both flower and vegetative buds. However, warm spells during late autumn and winter can lead to unfulfilled chilling requirements leading to erratic and delayed flowering. We hypothesized that storing plants at no light and low temperature could favor dormancy breaking and lead to early and synchronized flowering in response to forcing conditions in *C. japonica* ‘Nuccio’s Pearl’. Plants with fully developed floral primordia were stored at dark, 7°C, and RH > 90% for up to 8 weeks. To monitor endodormancy release during the storage, we evaluated the content of abscisic acid (ABA) in flower buds and the expression profiles of five putative genes related to dormancy and cold acclimation metabolism in leaves and flower buds. In addition, the expression of four anthocyanin biosynthesis pathway genes was profiled in flower buds to assess the effect of the treatment on flower pigment biosynthesis. At 0, 4, 6, and 8 weeks of cold treatment, 10 plants were transferred to the greenhouse and forced to flower. Forced plant flower qualities and growth were observed. The ABA content and the expression profiles of two dormancy-related genes (*CjARP* and *CjDEH*) suggested that dormancy breaking occurred after 6–8 weeks of cold treatment. Overall, plants treated for 6–8 weeks showed earlier vegetative sprouting, enhanced, and homogeneous flowering with reduced forcing time. Prolonged cold treatments also reduced flower size and longevity, anthocyanin content, and pigment biosynthesis-related gene transcripts. In conclusion, the cold treatment had a promotive effect on dormancy breaking but caused severe drawbacks on flower quality.

## Introduction

*Camellia japonica* L. (Theales, Theaceae) is an acidophilic evergreen shrub whose more than 3,000 cultivars (Mondal, 2011) are of great ornamental value. High quality marketable camellias require flowering to be abundant, early, and synchronized. Flower initiation and early differentiation of flower primordia start in late spring while flower bud development and visible bud enlargement sequel until autumn. At this point, like the majority of temperate woody plants (Okubo, 2000), camellias exhibit a well-defined dormancy or resting phase (Uemoto et al., 1990).

Bud dormancy or endodormancy is for most species induced by a decreasing photoperiod (Junttila, 1988). The release of flower bud dormancy is generally stimulated by extended periods at low temperatures. This is the case of genera phylogenetically close to *Camellia*, such as *Rhododendron* (Bodson, 1989; Christiaens et al., 2015) and *Hydrangea* (Anderson et al., 2009), where the exposure of floral buds to cold temperatures (between 2 and 7°C) stimulates endodormancy release and initiates normal growth and anthesis during the following spring (Arora et al., 2003). The length of the cold treatment needed for the resumption of growth is defined as the chilling requirement.

Abscisic acid (ABA) is involved in the induction and maintenance of endodormancy. The accumulation of ABA is generally observed at the onset of endodormancy, while as tissues switch from endodormancy to ecodormancy, ABA levels are generally known to decline (Horvath, 2009). In *Camellia*, endodormancy hormonal bases are represented by ABA, whose seasonal concentrations were reported to be high in deeply dormant vegetative buds and then reduced during dormancy release (Nagar, 1996). Dormancy leads also to changes in the expression of a large pool of genes (Horvath et al., 2003; Horvath, 2009). As emerged from recent studies concerning cold-induced and winter dormant *C. sinensis* (L.) Kuntze (Wang et al., 2009, 2014; Krishnaraj et al., 2011; Paul and Kumar, 2011, 2013), several genes responsible for the primary metabolism and stress responsive pathways are involved in the initiation, maintenance and release of dormancy, and can function as dormancy breaking markers. More in detail, some of them are commonly recognized as cold-regulated (*cor*) genes involved in cold acclimation (CAP *Arabidopsis cor* genes; Breton et al., 2003) or are known to activate *cor* genes themselves (AP2/ERF family transcription factor genes; Chinnusamy et al., 2007). Cold also up-regulates dehydrin genes (DEH) which are known to transcribe for cryoprotectors for cold-labile macromolecules (Hughes and Graether, 2011), while Auxin-Repressed Protein (ARP) genes have been found to be highly cold-inducible in *Arabidopsis* (Lee et al., 2005).

When dormancy requirements are fulfilled, the flower bud becomes a strong sink for assimilates. Sucrose synthase (SuSy) gene family has been found to encode for primary metabolism sucrose-cleaving enzymes whose up-regulation is expected in strong utilization sinks for sucrose (Sturm et al., 1999; Baud et al., 2004). In *Camellia*, the anthocyanin pathway will be up-regulated for flower pigmentation. Important genes in this pathway are the ones encoding for kaempferol 3-*O*-galactosyltransferase (F3GalTase), anthocyanidin reductase (ANR), dihydroflavonol 4-reductase (DFR), and flavonol synthase (FLS; Springob et al., 2003; Punyasiri et al., 2004; Montefiori et al., 2011).

Warm spells during late autumn and winter can lead to unfulfilled chilling requirements leading to erratic and delayed flowering, resulting in a poorly marketable product. In this study, we focused our attention on how controlled cold treatments could promote early and synchronized flowering and normal regrowth in dormant *C. japonica* ‘Nuccio’s Pearl’. In detail, we stored plants with fully differentiated and developed endodormant flower buds in a dark, low temperature regime for up to 8 weeks, which is standard practice for another acidophilic evergreen shrub, i.e., *Rhododendron simsii* (Christiaens et al., 2013, 2015). To monitor endodormancy release during the treatment, we evaluated the content of ABA in flower buds and the expression profiles of five putative genes related to dormancy and cold acclimation metabolism (*CjSuSy*, *CjERF*, *CjDEH*, *CjCAP*, and *CjARP*) in leaves and flower buds. In addition, the expression of four genes involved in the anthocyanin biosynthesis pathway (*CjF3GalTase*, *CjANR*, *CjDFR*, and *CjFLS*) was profiled in flower buds during the cold treatment, in order to assess the effect of prolonged cold on flower pigment biosynthesis. After the cold treatment, plants were forced to flower in the greenhouse and morphological characteristics were determined.

## Materials and Methods

### Plant Material and Growing Conditions

In February 2009, three rooted cuttings of *C. japonica* ‘Nuccio’s Pearl’ were potted into peat-based standard substrate (89% *Sphagnum* peat + 11% agriperlite), for a total of 82 pots. After re-potting, in June 2010, the camellias were sprayed twice with 1.5 l of flurprimidol at 15 mg l^-1^ (15–20 ml per plant averagely) to promote flower bud initiation and differentiation (Larcher et al., 2011). In mid-October, plants with fully developed floral primordia were stored at dark, 7°C, and RH > 90%. At 0, 4, 6, and 8 weeks of treatment, 10 plants were transferred to the greenhouse and forced to flower with semi-controlled temperature (night min 6°C, day min 12°C, night average 11°C, day average 15°C) and supplementary lighting (SON-T lamps, ±35 μmol m^-2^ s^-1^ for 8 h). During the forcing, mean RH was 75–80% on average, mean PAR (Photosynthetically Active Radiation) was 40.8 μmol m^-2^ s^-1^. The amount of chilling units (CU) was calculated according to the Utah Chill Unit model (Richardson et al., 1974). According to this model, 1 h at temperatures ranging from 2.5 to 9°C provides 1 CU and 1 h at temperatures from 9 to 12°C provides 0.5 CU. In addition, 1 h at a temperature between 16 and 18.5°C decreases the amount of accumulated CU by 0.5 units, while 1 h at temperatures higher than 18.5°C decreases the accumulated CU by 1 unit. Storing plants at 7°C for 4, 6, and 8 weeks of cold treatment provided 672, 1008, and 1344 CU, respectively. Day/night hourly temperatures at forcing conditions during the first 8 weeks from the start of the cold storage (**Supplementary Table S1**) were used to calculate the CU for control plants. Moreover, these temperature measurements were also used to adjust the CU values calculated for the plants that were transferred to forcing conditions after 4 and 6 weeks of cold storage. Adjusted CU values were 128, 888.5, 1178, and 1344, for 0, 4, 6, and 8 weeks of cold storage, respectively.

### Determination of Endogenous (±)ABA

Extraction and purification of (±)ABA was done as described previously (Chen et al., 1997) with minor modifications. After 0, 2, 4, 6, and 8 weeks of cold treatment, 12 flower buds were harvested from three plants. Each biological replicate represented the bulk of four buds randomly collected from the three plants. Immediately after excision, flower buds were freed of the outer scale and ground in liquid nitrogen. Approximately 500 mg of plant material was extracted in cold 80% aqueous methanol (5 ml/g FW) overnight at 4°C with ca 10 mg/l butylated hydroxytoluene to prevent oxidation. After centrifugation at 10,000 *g* (4°C, 20 min), the supernatant was filtered through a C18 Sep-Pak cartridge (Waters). The eﬄux was collected and dried in a stream of N_2_. The residue was dissolved in 1.5 ml phosphate-buffered saline (0.01 M, pH 9.2), adjusted to pH 8.5, and separated three times with an equal volume of ethyl acetate. The remaining water phase was adjusted to pH 2.5 and again extracted three times with ethyl acetate. The ethyl acetate phases were pooled and dried in a stream of N_2_. The residue was dissolved in 200 μl of 100% methanol and 50X diluted with tris-buffered saline (25 mM, pH 7.5). ABA levels were quantified by means of ELISA with a phytodetek-ABA kit (Agdia, Biofords, USA), following the manufacturer’s protocol. Two technical replicates were analyzed for each sample.

### Candidate Gene Isolation

A set of primers was designed for five dormancy-related and four flower color-related target genes (**Table 1**) using the web-based software Primaclade (The Kellogg Lab, University of Missouri), basing on multiple alignments of sequences lodged in GenBank belonging to *C. sinensis* and phylogenetically related species. Primer quality check for hairpin, self-dimer, and hetero-dimer formation was done using the online tool OligoAnalyzer 3.1 (Integrated DNA Technologies). In the same way, a set of primers was designed for four potential reference genes (**Table 1**) retrieved from a small pool of *C. japonica* sequences available in GenBank. The amplicon length was set to range from ∼80 to ∼170 bp, so as to ensure optimal polymerase efficiency and minimize the impact of the RNA integrity in RT-qPCR. The PCR assays were conducted in a total volume of 50 μl, containing 1 μl of cDNA (see the following paragraph), 10 μl of 5X Colorless GoTaq^®^ Reaction Buffer (Promega) with MgCl_2_, 0.5 μl of dNTPs (20 mM), 5 μl of each primer (10 μM) and 28.1 μl of mQ water. The PCR program was set as follows: 7 min at 94°C, 35 cycles of 94, 58, and 72°C for 45 s each, and a final 10 min extension step at 72°C. PCR products were checked by electrophoresis on a 1.2% agarose gel with the GeneRuler 100 bp Plus DNA Ladder (Fermentas, USA). After amplification, PCR products were cloned with the TOPO TA Cloning Kit (Invitrogen, USA) and four representative positive clones were submitted to direct colony PCR, purified (Werle et al., 1994), and sequenced following the protocol of the Big Dye Terminator v1.1 Cycle Sequencing Kit (Applied Biosystems, USA) with the ABI Prism^®^ 3130x/Genetic Analyzer (Applied Biosystems, USA). The sequences generated were validated using Blastx with minor modifications from default (no compositional adjustment and no filtering on low complexity regions) or Blastn (Altschul et al., 1997; Zhang et al., 2000) against the swissprot and the refseq database or the non-redundant nucleotide GenBank database, respectively.

**Table 1:** Primer pairs (5′–3′) used for isolation and RT-qPCR assay for candidate target (T) and reference (R) genes.

Candidate gene	Type	Forward 5′–3′	Reverse 5′–3′	Length (bp)
*CjARP*	T	GTGTGGAGGAGCGTGTTC	GCTCCTCGTCTCCCCACT	138
*CjSuSy*	T	CCAAGGATTTGGAAGAGCAA	TTCCTCACCCTGTTCATCTG	112
*CjCAP*	T	GGATCGGACAAATTGGAAGAC	TTTAGGGAAGAAGAGGCG	154
*CjDEH*	T	GCCACACCACCACTAATAA	AACGCATTTATGACAACACA	134
*CjERF*	T	TAAATGGGCTGCTGAGAT	ACCTTAGCTTTCTTGCCAC	132
*CjF3GalTase*	T	CGACATGGCAGAGGAGA	AATCCTGGGACGAATTT	159
*CjANR*	T	TGTCCCTACTGATTTTGGAG	TGAAACTGAATCCCTCTTTG	86
*CjDFR*	T	AAGAGGCTGGTGTTCACAT	TCCCATGCTGCTTTCTCTGC	167
*CjFLS*	T	AGAATGAACAACCTGCAATC	TTACAACCTGAAATATCCCC	144
*CjATPSβ*	R	AACCCACCCTTAGTACCGAAAT	TGCAGGTACATAAACTGCTTGAA	98
*CjNADH5*	R	AGCAAGGTGCTCCCCTTTA	GTTGCCGCAAGGAATGAC	90
*CjRNAPβ*	R	GCTACGAAAATCTTTGTCAATGG	TACCCGTCTCCTCAGTCGTC	93
*CjRS3P*	R	CCTTCAGAAAAACGAAGTTATTCAA	CAGGGAGGGTCCTTTTAAGTAGA	93


*The length of the amplicon is reported.*

### Sample Preparation and RT-qPCR Assay

Two biological replicates, each consisting of two mature leaves or two flower buds harvested from a single but different plant per replicate, were sampled every one (for leaves) or two weeks (for flower buds) during the treatment and flash-frozen in liquid nitrogen. Flower buds and leaves, freed of the outer scale and the petiole, respectively, were finely ground in an RNase-free area and stored at –80°C. Extraction of total RNA was done according to the CTAB method (Doyle and Doyle, 1987) with minor modifications on approximately 75 mg of plant material. DNase treatment was performed with the DNA-*free* kit (Ambion) on 80 μl of eluted RNA by adding 10 μl of DNaseI buffer and 1.5 μl of rDNaseI and incubating for 30 min at 37°C. Ten microliter of DNase Inactivation Reagent was added and after an incubation of 2 min at room temperature and a centrifugation (90 s at 10,000 *g*), the supernatant was transferred to a new tube. The samples were then purified by adding 0.3 M Sodium Acetate pH 5.5 and two and a half volumes of 100% EtOH and storing overnight at –20°C. The supernatant was removed after 25 min centrifugation (18,200 *g*, 4°C) and 1 ml 70% EtOH was added. Again tubes were centrifuged for 20 min at the same conditions and the supernatant was discarded. The RNA pellet was dried in a vacuum-desiccator and dissolved in 25 μl of RNase-free water. Total RNA was quantified using a NanoDrop 1000 spectrophotometer (Isogen, The Netherlands) and samples were stored at –80°C. The synthesis of cDNA was performed according to the laboratory protocol (De Keyser et al., 2013) using the SuperScript^TM^ III First-Strand Synthesis SuperMix (Invitrogen, USA) with Oligo(dT)_20_ for priming on 1350 ng of RNA. As a control for gDNA residue contamination, noRT samples were created in the same way as cDNA samples, except for the reverse transcriptase that was not added. Standard pDNA curves for the assessment of gene specific PCR efficiencies were also considered during each assay (De Keyser et al., 2013). The plasmid DNA for all candidate target and reference genes was purified using the PureLink^TM^ HQ Mini plasmid Purification Kit (Invitrogen) and linearized using 10 U of HindIII (Invitrogen) for 2 h at 37°C, followed by an enzyme inactivation step of 10 min at 70°C. The stock concentration of plasmids was diluted to a working solution of 1 ng/μl in 50 ng/μl yeast tRNA (Invitrogen). Thus, standard curves were constructed as six serial 10X dilutions of this working solution in yeast tRNA (50 ng/μl). The dilutions were stored at 4°C and used within a few hours. The cDNA and the noRT samples were 4X diluted and, together with water control (NTC) and standard curves, were jointly measured in technical duplicate in a LightCycler480 (Roche). The assays were conducted in a white 384-wellplate (Roche) sealed with adhesive film, in a total volume of 10 μl, containing 375 nM of each primer (**Table 1**), 5 μl of LightCycler480 SYBR Green I Master (Roche), and 2 μl of sample. Cycling conditions were 5 min at 95°C, followed by 40 cycles of 10 s 95°C, 12 s 60°C, and 10 s 72°C. Data acquisition was done at the end of every cycle. Melting curve analysis was performed as follows: 5 s 95°C, 1 min 65°C, and heating to 97°C with a ramp rate of 0.06°C/s. Data acquisition occurred 10 times for every 1°C raise. Data were analyzed using the LightCycler480 software version 1.5 (Roche). Quantification cycle (Cq; Bustin et al., 2009) values were calculated by means of the second derivative maximization method and exported to qbase^+^ (Hellemans et al., 2007; Biogazelle, Belgium) to select the most appropriate reference genes using the GeNorm application (Vandesompele et al., 2002). The calibrated normalized relative quantities (CNRQ) of gene expression were calculated based on gene specific amplification efficiencies (derived from standard curves) and a normalization factor based on the geometric mean of the validated reference genes. Prior to statistical analysis, CNRQ values were log-transformed.

### Assessment of Flowering Quality and Growth

Flowering was monitored on four groups of 10 camellia plants each, stored for 0, 4, 6, and 8 weeks at 7°C at 3–4 day-time interval. A number of surveys were carried out during the entire forcing phase in order to evaluate flowering and growth. During the whole flowering period, the number of flowers produced per plant and their longevity (days from early opening to fall) were observed. Moreover, for every full bloom, the flower conical volume was measured three times during the anthesis, according to the following formula: π × (FØ ÷ 2)^2^ × FD ÷ 3 (with *F*Ø being flower diameter and *FD* representing flower depth; Larcher et al., 2011). On each group of plants, the duration of flowering was determined as the days between the first anthesis and 100% flowering of the whole group of 10 plants, and the forcing need as the days between the start of forcing and 25% flowering of the whole group of 10 plants. Twenty weeks after the beginning of the experiment, when control and treated plants were in full bloom, the percentage of sprouting vegetative buds was measured. At the same time, the anthocyanin content of flower petals was assessed in three biological replicates per group of plants, according to a protocol developed for tomato seedlings (Adamse et al., 1989) with minor modifications, using cyanidin chloride (Roth, Karlsruhe, Germany) as a standard.

### Statistical Analyses

The effect of the cold treatment on flower bud ABA content, flowering quality and growth-related parameters were determined by means of the parametric analysis of variance (ANOVA) or the non-parametric Kruskal–Wallis ANOVA, according to data distribution. ANOVAs were followed by *post ho*c tests using the Ryan–Einot–Gabriel–Welsch (REGW-*F*) test for parametric data or the Mann–Whitney *U* test with Bonferroni correction for non-parametric data. The Pearson’s correlation was assessed between all the combinations of the parameters measured during the treatment and the following forcing stage. All analyses were run using SPSS statistical package version 19.0.

## Results

### ABA Levels during the Cold Treatment

**FIGURE 1:**
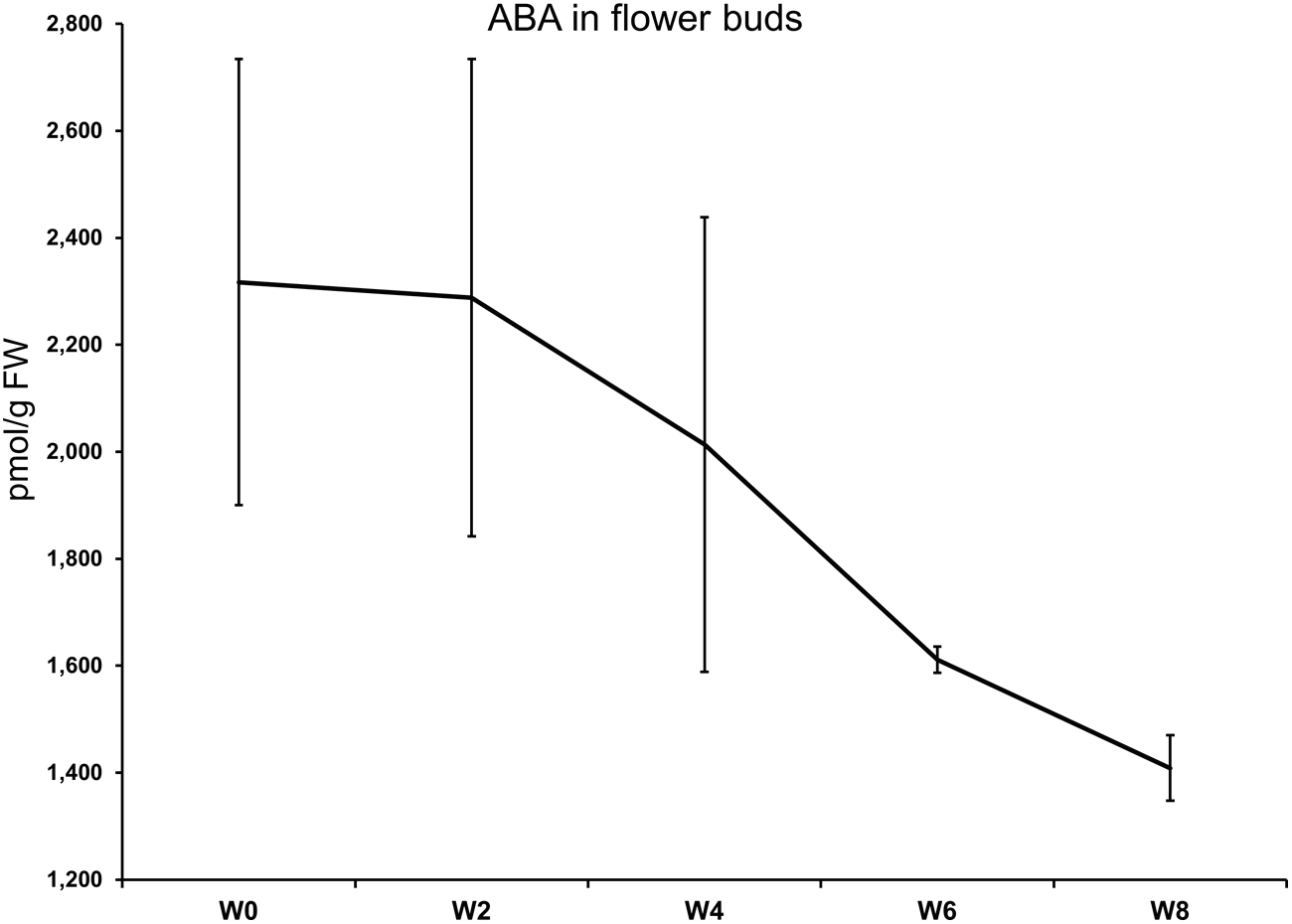
**Abscisic acid (ABA) levels in flower buds (*y*-axis) after 0, 4, 6, and 8 weeks at 7°C + dark (W0–W8).** Bars correspond to the SEM.

The levels of ABA in flower buds after 0, 2, 4, 6, and 8 weeks of cold treatment are shown in **Figure 1.** No statistical differences were highlighted between the timepoints by the ANOVA for unequal variances (Welch *F* test; *p*-value = 0.1691). The highest ABA concentration (2317 pmol/g FW) was measured at the start of the cold treatment. At week 6, a clear-cut drop was registered while the lowest value was seen at week 8, where ABA average concentration was 1409 pmol/g FW. The ABA downward trend was accompanied by a marked reduction in the heterogeneity of the biological replicates at week 6 and 8 (see the SEM; **Figure 1**).

### Gene Expression Profiling during the Cold Treatment

Thirteen candidate gene fragments were successfully amplified from cDNA samples and sequenced (**Supplementary Data Sheet 1**). The fragment length ranged from 86 to 167 bp. Blastx confirmed homology with orthologous genes from other species (**Table 2**). In one case (*CjDEH*), the amplicon was ∼85% non-coding DNA, thus, its identity was validated through a Blastn search against the non-redundant nucleotide database. Blastx and Blastn searches returned high query coverage (93–100%) and a medium to high level of homology (65–100%) with referenced sequences.

**Table 2:** Homology with swissprot, refseq, or the non-redundant nucleotide database accessions according to Blastx with minor modifications from default (no compositional adjustment and no filtering on low complexity regions) or Blastn (default).

Candidate gene	Homologous gene	Acc. No.	*e*-value	Query cov. (%)	ID (%)	Species
*CjARP*	Auxin-repressed 12.5 kDa protein	sp| Q05349.1	3*e*-18	100	77	*Fragaria x ananassa*
*CjSuSy*	Sucrose synthase isoform 1	sp| P49035.1	3*e*-15	96	89	*Daucus carota*
*CjCAP*	Cold-regulated 413 plasma membrane protein 1	sp| Q9XIM7.1	5*e*-22	99	73	*Arabidopsis thaliana*
*CjDEH*	Dehydrin	gb| FJ436978.1	3*e*-58	100	99	*Camellia sinensis*
*CjERF*	Ethylene-responsive transcription factor 1	sp| Q6K7E6.1	9*e*-21	97	95	*Oryza sativa*
*CjF3GalTase*	Kaempferol 3-*O*-beta-D-galactosyltransferase	sp| Q9SBQ8.1	2*e*-17	98	65	*Petunia x hybrida*
*CjANR*	Anthocyanidin reductase	ref| XP_010241022.1	5*e*-08	97	93	*Nelumbo nucifera*
*CjDFR*	Dihydroflavonol-4-reductase	sp| P51102.2	2*e*-25	98	80	*Arabidopsis thaliana*
*CjFLS*	Flavonol synthase/flavanone 3-hydroxylase	sp| Q9M547.1	6*e*-14	97	70	*Eustoma exaltatum*
*CjATPS*β	ATP synthase subunit beta	sp| Q0G9V5.1	5*e*-13	97	100	*Daucus carota*
*CjNADH5*	NADH-ubiquinone oxidoreductase chain 5	sp| Q37680.1	2*e*-10	96	97	*Triticum aestivum*
*CjRNAP*β	DNA-directed RNA polymerase II subunit 2	sp| P38420.2	1*e*-09	100	94	*Arabidopsis thaliana*
*CjRS3P*	Ribosomal protein S3	sp| P27754.2	2*e*-10	93	100	*Oenothera berteroana*


*Name of homologous gene, accession number, e-value, query coverage, identity (ID), and species of origin are reported.*

RNA absorbance ratios were optimal (**Supplementary Table S2**). The melting curves of the target and reference gene amplicons showed only single peaks and no significant noise due to primer dimers. For all genes, noRT amplification was controlled in every sample by looking at the *C*q-value and melting curve analysis. All noRT amplicon *C*q-values showed more than five cycles of difference with their related RT samples and contamination was therefore considered negligible (Hellemans et al., 2007). Occasionally, noRT sample amplicons showed a shifted melting curve, potentially due to marginal gDNA leftover amplification. In qbase^+^, target gene expression values were calculated using gene specific standard curve-derived efficiencies (**Supplementary Table S3**). A combination of three validated reference genes was used for normalization for both flower bud samples (*CjRPBS2*, *CjNADH5*, *CjRS3P*; *M*-value = 0.774 and Coefficient of Variation = 0.313) and leaf samples (*CjATPS*β, *CjNADH5*, *CjRS3P*; *M*-value = 0.614; Coefficient of Variation = 0.273).

**FIGURE 2:**
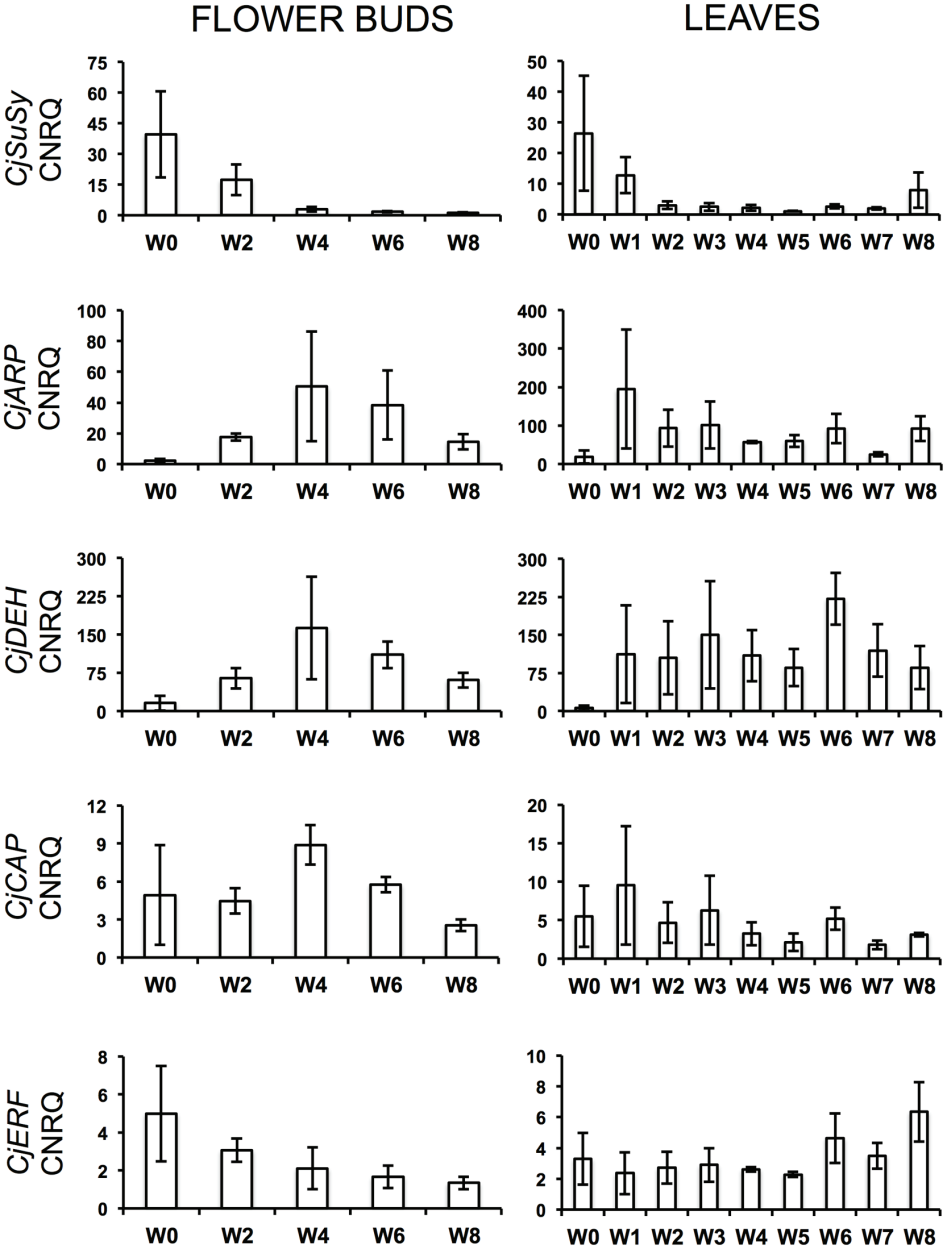
**Calibrated normalized relative quantities (CNRQ) of flower bud **(Left)** and leaf **(Right)** transcripts of dormancy-related genes during the weeks of treatment at 7°C + dark (W0–W8).** Bars correspond to the SEM.

**FIGURE 3:**
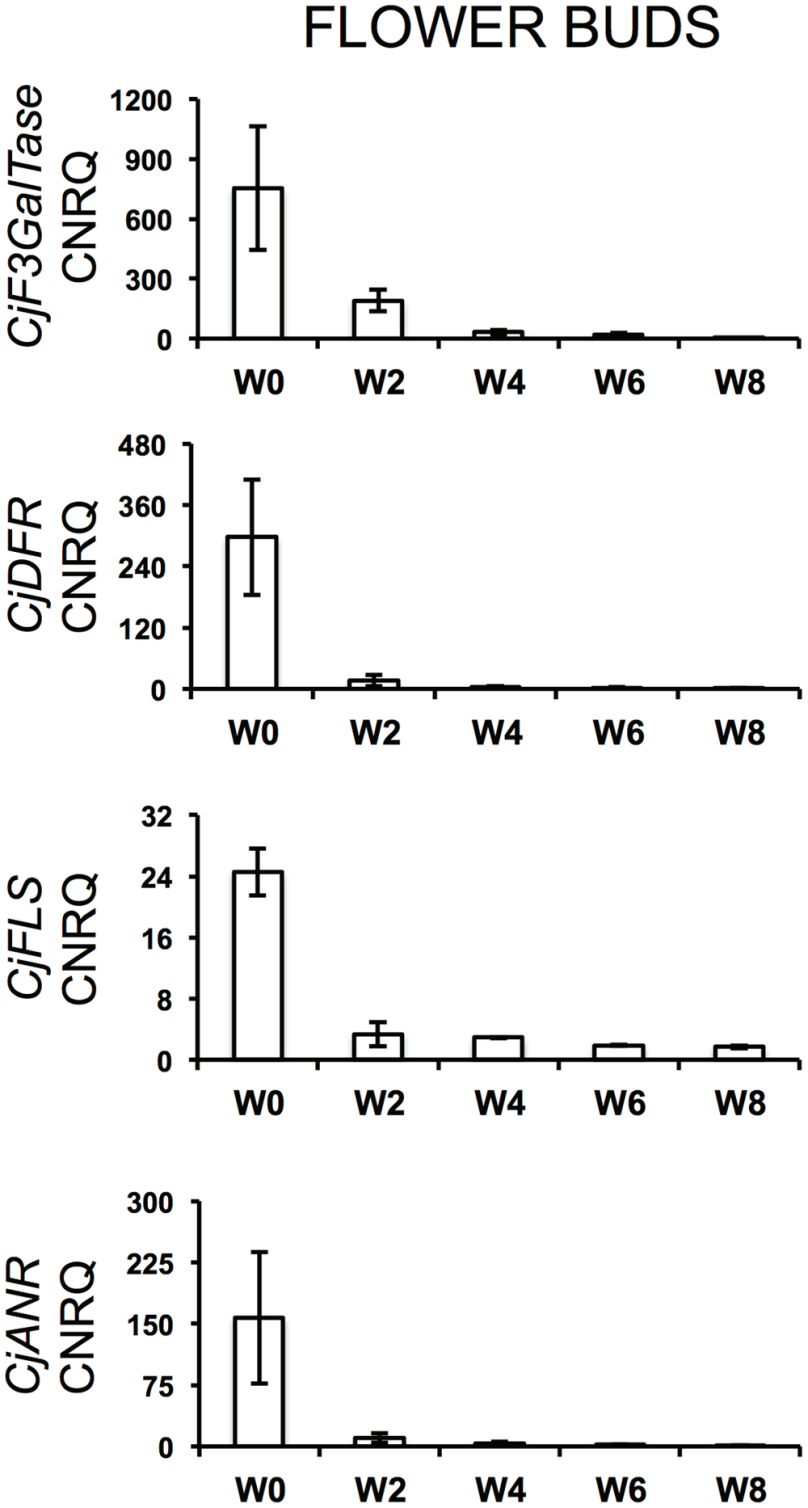
**Calibrated normalized relative quantities of flower bud transcripts of flower color-related genes during the weeks of treatment at 7°C + dark (W0–W8).** Bars correspond to the SEM.

During the cold treatment, *CjSuSy* expression patterns followed a downward trend in both flower buds and leaves (**Figure 2**). Only in leaves, a new slight increase was registered after 8 weeks of treatment. *CjARP*, *CjDEH*, and *CjCAP* expression in flower buds (**Figure 2**) generally followed the same trend, with expression peaks after 4 weeks of treatment, although large standard errors were present, and down-regulation at week 0 and 8. The only exception was found in *CjCAP* expression at week 0, where a high CNRQ value with large standard error was measured. In leaves (**Figure 2**), both *CjARP* and *CjDEH* transcripts were up-regulated during the treatment, while *CjCAP* showed no trend. *CjERF* transcripts in flower buds exhibited a slight gradual down-regulation during the treatment, while the opposite trend was detected in leaves, with an expression peak after 8 weeks of treatment. Flower color-related genes (*CjF3GalTase*, *CjANR*, *CjDFR*, and *CjFLS*) showed a clear-cut down-regulation immediately after camellias were transferred to cold treatment conditions (**Figure 3**).

### Plant Morphological Response

The plant morphological parameters observed during the forcing are displayed in **Table 3.** The number of flowers produced per plant during the forcing phase showed an increase with prolonged cold treatment, although no statistical significance was attributed to this variation. The ratio between the number of buds that successfully completed the anthesis and the number of buds that started the opening process (anthesis completion ratio) showed a significant increase in plants treated for 8 weeks. Flowering was accomplished 7–10 days faster in cold treated plants than in non-treated plants. The number of days at forcing conditions needed to achieve 25% of flowering (forcing need) decreased with increasing weeks of treatment. Anyway, the total number of days needed from the beginning of the dark/cold treatment to achieve 25% of flowering was almost unchanged (data not shown). Cold treated plants produced flowers with a significantly smaller volume, with the lowest value registered in camellias treated for 8 weeks (67.88 cm^3^). The anthocyanin levels in the petals showed a significant decrease when cold treated for 6 and 8 weeks. Flower longevity significantly decreased by ∼10 days in plants treated for 4 weeks or more. Vegetative sprouting occurred significantly earlier in plants treated for 6–8 weeks.

**Table 3:** Effects of the cold treatment on flowering and vegetative regrowth.

Weeks at 7°C + dark	N. of flowers per plant	Anthesis completion ratio	Duration of flowering (days)	Forcing need (days)	Flower volume (cm^3^)	Anthocyanin (μmol/g FW)	Flower longevity (days)	Sprouting vegetative buds (%)
0	2.1	0.57a^1^	80	114	106.8c	0.240b	24.14b	0.0a
4	2.3	0.80ab	73	93	91.1b	0.256b	15.74a	1.3a
6	2.3	0.87ab	70	72	72.5a	0.195a	14.78a	42.1b
8	2.9	0.95b	70	57	67.9a	0.174a	14.14a	64.5b
*p*-value	ns	^∗∗^	nd	nd	^∗∗∗^	^∗∗^	^∗∗∗^	^∗∗∗^


*For the number of flowers per plant, only fully opened blooms were considered. ^1^Means followed by the same letter do not differ significantly at *P* < 0.05, according to REGW-*F* test for parametric data or the Mann–Whitney *U* test with Bonferroni transformation for non-parametric data. The statistical significance (*p*-value, ^∗∗^*p* < 0.01, ^∗∗∗^*p* < 0.001, ns, non-significant, nd, not determinable) of between-subjects effects’ tests is provided.*

### Correlations among Gene Expression, ABA Level, Plant Morphology, and Treatment Duration

The correlations between dormancy- and cold-related gene expression levels, treatment duration, ABA, and plant morphology are shown in **Table 4.** The level of transcripts of *CjDEH* and *CjARP* was positively correlated during the treatment. *CjSuSy* and *CjERF* transcript levels were also positively correlated and both genes were shown to correlate their expression negatively with the duration of the treatment and the anthesis completion ratio, and positively with the duration of flowering and flower longevity. The transcription level of *CjERF* was also positively correlated to forcing need and flower volume. ABA levels were positively correlated to the transcription of these two genes, the forcing need, and flower volume, and negatively correlated to the length of the treatment, the anthesis completion ratio, and the percentage of sprouting vegetative buds 20 weeks after the beginning of the experiment.

**Table 4:** Significant Pearson’s correlation coefficients among dormancy-related candidate gene expression profiles and between these and the duration of the cold treatment and morphometric parameters.

Correlates			*R*	*p*-value	*N*	Significance
*CjDEH*	vs.	*CjARP*	0.996	0.0003	5	^∗∗∗^
*CjERF*	vs.	Weeks of treatment	-0.984	0.0024	5	^∗∗^
		*CjSuSy*	0.981	0.0032	5	^∗∗^
		Anthesis completion ratio	-0.998	0.0020	4	^∗∗^
		Duration of flowering	0.989	0.0113	4	^∗^
		Flower longevity	0.983	0.0174	4	^∗^
		Forcing need	0.953	0.0475	4	^∗^
		Flower volume	0.957	0.0434	4	^∗^
*CjSuSy*	vs.	Weeks of treatment	-0.961	0.0091	5	^∗∗^
		Anthesis completion ratio	-0.984	0.0158	4	^∗^
		Duration of flowering	0.991	0.0087	4	^∗∗^
		Flower longevity	0.998	0.0026	4	^∗∗^
Abscisic acid	vs.	Weeks of treatment	-0.972	0.0057	5	^∗∗^
		Anthesis completion ratio	-0.954	0.0456	4	^∗^
		Forcing need	0.998	0.0020	4	^∗∗^
		Flower volume	0.994	0.0062	4	^∗∗^
		Sprouting vegetative buds	-0.954	0.0456	4	^∗^
		*CjSuSy*	0.919	0.0274	5	^∗^
		*CjERF*	0.920	0.0269	5	^∗^


*The statistical significance (^∗^*p* < 0.05, ^∗∗^*p* < 0.01, ^∗∗∗^*p* < 0.001) of the Pearson’s correlation is provided.*

The correlations between flower color-related gene expression levels, treatment duration, ABA, and flowering are shown in **Table 5.** The expression of all flower color-related candidate genes was positively correlated to the one of *CjERF*. Flower color-related gene expression profiles were mostly positively correlated to each other (*CjANR*–*CjDFR*, *CjF3GalTase*–*CjDFR*, *CjF3GalTase*–*CjANR*, *CjFLS*–*CjANR*, and *CjFLS*–*CjDFR*). The expression of *CjANR*, *CjF3GalTase*, *CjDFR*, and *CjFLS* was negatively correlated to the anthesis completion ratio. The levels of *CjANR*, *CjF3GalTase*, and *CjDFR* were correlated positively to the expression of *CjSuSy* and negatively to the duration of the cold treatment. *CjANR*, *CjDFR*, and *CjFLS* levels were positively correlated to the duration of flowering and flower longevity. Finally, *CjF3GalTase* transcription level correlated positively to the level of ABA and to the forcing need.

**Table 5:** Significant Pearson’s correlation coefficients among flower color-related candidate gene expression profiles and between these and the duration of the cold treatment, morphometric parameters, and dormancy-related candidate gene expression profiles.

Correlates			*R*	*p*-value	*N*	Significance
*CjANR*	vs.	Weeks of treatment	-0.910	0.0318	5	^∗^
		*CjERF*	0.968	0.0067	5	^∗∗^
		*CjSuSy*	0.942	0.0167	5	^∗^
		*CjF3GalTase*	0.890	0.0429	5	^∗^
		*CjDFR*	0.998	0.0001	5	^∗∗∗^
		*CjFLS*	0.983	0.0026	5	^∗∗^
		Anthesis completion ratio	-0.970	0.0305	4	^∗^
		Duration of flowering	0.984	0.0161	4	^∗^
		Flower longevity	1.000	0.0000	4	^∗∗∗^
*CjF3GalTase*	vs.	Weeks of treatment	-0.986	0.0021	5	^∗∗^
		*CjDFR*	0.913	0.0302	5	^∗^
		*CjERF*	0.967	0.0073	5	^∗∗^
		*CjSuSy*	0.943	0.0163	5	^∗^
		Anthesis completion ratio	-0.979	0.0209	4	^∗^
		Forcing need	0.963	0.0370	4	^∗^
		Abscisic acid	0.943	0.0163	5	^∗^
*CjDFR*	vs.	Weeks of treatment	-0.929	0.0223	5	^∗^
		*CjERF*	0.980	0.0035	5	^∗∗^
		*CjFLS*	0.974	0.0050	5	^∗∗^
		*CjSuSy*	0.956	0.0109	5	^∗^
		Anthesis completion ratio	-0.977	0.0235	4	^∗^
		Duration of flowering	0.984	0.0162	4	^∗^
		Flower longevity	1.000	0.0005	4	^∗∗∗^
*CjFLS*	vs.	*CjERF*	0.928	0.0229	5	^∗^
		Anthesis completion ratio	-0.976	0.0239	4	^∗^
		Duration of flowering	0.992	0.0080	4	^∗∗^
		Flower longevity	0.998	0.0016	4	^∗∗^


*The statistical significance (^∗^*p* < 0.05, ^∗∗^*p* < 0.01, ^∗∗∗^*p* < 0.001) of the Pearson’s correlation is provided.*

The correlations among plant morphological parameters are shown in **Table 6.** The duration of the treatment was positively correlated to the anthesis completion ratio, and correlated negatively to the duration of flowering, the forcing need and flower longevity and volume. The anthesis completion ratio was negatively correlated to the duration of flowering, forcing need, flower volume and longevity. Positive correlations were also found between the duration of flowering and flower volume and longevity, and between forcing need and flower volume. Finally, anthocyanin petal content was negatively correlated to the percentage of sprouting vegetative buds after 20 weeks from the beginning of the experiment.

**Table 6:** Significant Pearson’s correlation coefficients among morphometric parameters and between these and the duration of the cold treatment.

Correlates			*R*	*p*-value	*N*	Significance
Anthocyanin	vs.	Sprouting vegetative buds	-0.981	0.0195	4	^∗^
Anthesis completion ratio	vs.	Weeks of treatment	0.993	0.0067	4	^∗∗^
		Duration of flowering	-0.980	0.0204	4	^∗^
		Forcing need	-0.966	0.0336	4	^∗^
		Flower volume	-0.963	0.0367	4	^∗^
		Flower longevity	-0.970	0.0300	4	^∗^
Duration of flowering	vs.	Weeks of treatment	-0.962	0.0380	4	^∗^
		Flower volume	0.955	0.0450	4	^∗^
		Flower longevity	0.983	0.0168	4	^∗^
Forcing need	vs.	Weeks of treatment	-0.989	0.0107	4	^∗^
		Flower volume	0.990	0.0103	4	^∗^
Flower volume	vs.	Weeks of treatment	-0.980	0.0196	4	^∗^


*The statistical significance (^∗^*p* < 0.05, ^∗∗^*p* < 0.01) of the Pearson’s correlation is provided.*

## Discussion

Camellia buds showed a deep endodormancy, as it took 114 days of forcing conditions for non-treated buds to achieve 25% flowering. This could be partly due to plant growth regulator application which tend to increase the level of dormancy and thereby lead to a higher cold demand, as confirmed by studies on azalea (Christiaens et al., 2012b). *C. japonica* ‘Nuccio’s Pearl’ did not exhibit an absolute cold requirement for breaking dormancy, since it was able to flower without being treated for long periods at uninterrupted low temperatures. Although control plants occasionally experienced dormancy-breaking temperatures during the first 8 weeks of the experiment, only minor chilling (128 CU) was accumulated during this time.

Although negatively correlated to the duration of the treatment, ABA content in flower buds was still quite high after 4 weeks (>2000 pmol/g FW), as seen in the late azalea cultivar ‘Mw. Kint’ (Christiaens et al., 2013). The strong drop measured after 6 weeks of treatment coincided with a lower variance of ABA content in the biological replicates, suggesting that dormancy release was present in all buds and this is also shown by the increase in flowering homogeneity in plants treated for 6 and 8 weeks. Similarly, in another study, a decrease of ABA levels was observed in flower bud tissues of *Rhododendron* L. ‘Prize’ after 6 weeks at 9°C (Pemberton et al., 1985). The effect on dormancy release was confirmed by the plant morphological response to the treatment. Direct consequences of the lower level of endodormancy in flower buds were seen on other flowering characteristics, such as flower production, higher anthesis completion ratio, and the reduced forcing time needed to initiate flowering. More florets per flower, a higher percentage of flowering, and an earlier flowering were also found in *Delphinium* seedlings treated with cold (5–10°C) compared to non-treated seedlings (Ogasawara et al., 2001). Similarly, in *Hydrangea* (Wallerstein and Rünger, 1985), peony (Fulton et al., 2001), and *Helleborus* (Christiaens et al., 2012a) the required days of forcing depended on the extent of the cold treatment.

Prolonged dark cold treatment produced also a series of drawbacks on individual flower quality. Petal anthocyanin content, flower volume, and flower longevity were reduced at different rates in treated plants. The decrease in anthocyanin content could have been caused by the dark conditions during the cold treatment, which prevented flower buds from getting for an extended period (1–2 months) enough visible and UV light stimuli that are known to trigger anthocyanin biosynthesis (Dooner et al., 1991). This is consistent with flower color-related gene expression, which suffered a severe down-regulation immediately after the beginning of the treatment, which was maintained during the cold treatment. Flower-color related genes might have resumed their activity when plants were placed in the greenhouse at forcing conditions, however, we highlighted that anthocyanin accumulation in petals was compromised after 6 or more weeks of cold treatment (**Table 3**). Moreover, keeping plants in dark conditions might have caused, on the long run, carbon starvation. Starch, sucrose, raffinose, fructose, and glucose showed to decrease, sometimes dramatically, in the late-flowering azalea *Rhododendron simsii* ‘Mw. G. Kint’ during increasing weeks at dark and 7°C (Christiaens et al., 2015). The authors related this global carbon depletion to low leaf starch content at the start of the cold treatment. If this was the case for *C. japonica* ‘Nuccio’s Pearl’, a negative effect on the sucrose-specific induction of the biosynthetic pathway of anthocyanins (Solfanelli et al., 2006) might have also been triggered. Similar anthocyanin biosynthesis suppression by dark conditions and lack of sugar was described in a cell suspension culture of *Rosa hybrida* ‘Charleston’ (Hennayake et al., 2006). In contrast, prolonged cold storage was shown not to be a problem for flower quality in azalea (Christiaens et al., 2013). Low availability of metabolizable sugars could also have negatively affected cell elongation, bud size and final flower volume. Moreover, the enhanced dormancy release of vegetative buds of plants treated for 4 weeks or more could have contributed to the quality drawback on flowers. This overlap with the flowering process might have overloaded the plant, already depleted in sugar, with too many sinks in need of nutrient support, this way interfering with the correct progress of anthesis and causing negative effects on flower quality (volume and longevity). Accordingly, the strong decrease of *CjSuSy* in flower bud and leaf tissues during the treatment could indicate a down-regulation caused by sucrose depletion (Koch et al., 1992). The successfully isolated partial gene *CjSuSy* had the highest similarity with the *Daucus carota* SuSy isoform 1 that encodes for a sucrose-cleaving enzyme that provides UDP-glucose and fructose and is expressed in several tissues, including flower buds (Sebková et al., 1995; Sturm et al., 1999). In *Arabidopsis*, the induction of several SuSy isoforms has been found under conditions of increased demand for translocation of carbohydrates such as O_2_ deficiency, dehydration, osmotic stress, and cold treatment (Baud et al., 2004). However, SuSy isoforms are coded by a multigene family and are supposed to be largely responsible for both sucrose breakdown and accumulation (Moriguchi et al., 1992; Huber and Huber, 1996; Schrader and Sauter, 2002; Coleman et al., 2009; Fujii et al., 2010). In *C. sinensis*, Paul and Kumar (2011) highlighted a down-regulation of SuSy (*CsSuSy*) expression during winter dormancy in the apical bud and two subtending leaves, similarly to our findings. However, these authors have carried out a seasonal monitoring and no cold/dark treatment was applied. The successfully isolated partial gene *CjERF* was highly similar to *OsEREBP1* (rice), a transcriptional activator involved in defense signaling pathway (Cheong et al., 2003), belonging to the AP2/ERF family transcription factors that is generally induced by cold as seen in *Arabidopsis* (Thomashow, 2010), rice (Tian et al., 2011), and *C. sinensis* (Krishnaraj et al., 2011). Their main role is to bind to *cis*-elements in the promoters of *cor* (cold-regulated) genes and activate their expression (Chinnusamy et al., 2007). The isolated partial gene *CjCAP* was homologous to an *Arabidopsis cor* gene, *AtCOR413-PM1*, involved in cold acclimation (Breton et al., 2003). A cold acclimation protein gene, *CsCOR413*, was found to be up-regulated during winter dormancy in *C. sinensis* (Paul and Kumar, 2011). In particular, it was shown to exhibit up-regulation upon exposure to low temperatures in both dormant and actively growing tissues. In addition, the authors found that the presence of ABA at low temperatures led to quicker up-regulation of these genes in dormant tissues as compared with the tissues at low temperatures but without ABA, suggesting the involvement of this hormone in the sensitivity of tissues to cold. *CjERF* expression in flower buds was up-regulated before the start of the treatment and positively correlated to ABA downward trend. This could suggest an ABA-like role for *CjERF* in preparing plant tissues to low temperatures. *CjCAP* transcripts in flower buds showed indeed a peak of expression after 4 weeks of treatment, demonstrating that camellias were prepared to enact cold acclimation. Similarly, *CjARP* and *CjDEH* were clearly induced by cold and showed, in flower buds, the peak of their highly correlated expression at the same timepoint of the treatment as *CjCAP*. The three genes together showed also a joint decrease at week 8, putatively signaling endodormancy release. ARP genes have been found highly transcribed at dormant stages and low at sprouting in tea (Wang et al., 2014). Their induction by cold has been described in many other species, including *Arabidopsis* (Lee et al., 2005), *Brassica rapa* (Lee et al., 2013), *Cicer arietinum* (Mantri et al., 2007), *Robinia pseudoacacia* (Park and Han, 2003), and in the calcifuge *Vaccinium corymbosum* (Dhanaraj et al., 2007). Specific combinations of genes coding for dehydrins are also induced by cold or drought stress (Thomashow, 1998; Tommasini et al., 2008) as these proteins function as cryoprotector for cold-labile macromolecules (Hughes and Graether, 2011). Similarly to our study, two putative tea dehydrin genes, *CsDEH1* and *CsDEH2*, were shown to be up-regulated during winter dormancy and down-regulated during dormancy release and active growth, exhibiting strong up-regulation upon exposure to low temperatures in both dormant and actively growing tissues, particularly after exogenous ABA application, as seen for *CsCOR413* (Paul and Kumar, 2011, 2013). Another tea putative dehydrin gene was also demonstrated to stay up-regulated at bud dormancy and shows less accumulation during bud sprouting (Wang et al., 2014).

The present study showed that flower bud dormancy was affected by the cold treatment. *C. japonica* ‘Nuccio’s Pearl’ did not exhibit an absolute cold requirement for breaking dormancy. However, our results indicated that 6–8 weeks of cold storage had a promotive effect on dormancy breaking. The response to these treatments could be resumed in an earlier vegetative sprouting, an increased percentage of flowers that could successfully complete the anthesis process, a lower need for forcing and a more uniform flowering. The reduced ABA levels in flower buds were linked to the release of endodormancy. *CjARP* and *CjDEH* expression profiles acted as suitable markers for endodormancy breaking. The cold treatments showed also some drawbacks on flower quality. These included a reduction in flower size, anthocyanin content and flower longevity. We suggest that the occurrence of dark-induced respiration at the expense of flower bud sugar accumulation during the treatment could be the leading cause. Future studies should concentrate on how to overcome these quality-related problems, e.g., by carrying out the cold treatment at an earlier bud developmental stage to avoid interference with floral pigmentation, and supplying a photoperiod and slightly higher dormancy-breaking temperatures to stimulate photosynthesis.

## Author Contributions

VS, M-CV, ED, and AB contributed to the conception and design of the work. AB, AC, and ED carried out laboratory and/or statistical analyses. AB drafted the manuscript that was critically revised and approved by VS, M-CV, ED, and AC.

## Conflict of Interest Statement

The authors declare that the research was conducted in the absence of any commercial or financial relationships that could be construed as a potential conflict of interest.
