# Supplementary material for: Cold Treatment Breaks Dormancy but Jeopardizes Flower Quality in Camellia japonica L
Source: Front Plant Sci. 2015 Nov 12;6:983. doi: 10.3389/fpls.2015.00983 (PMC4641915; doi:10.3389/fpls.2015.00983)
Supplement: Supplementary file 2 [file Table_2.DOCX]

***Supplementary Material***

**Cold treatment breaks dormancy but jeopardizes flower quality in *Camellia japonica* L.**

Berruti A.^1,2^, Christiaens A.^3^, De Keyser E. ^4^, Van Labeke M.C.^3^, Scariot V.^2^

**Corresponding author:**

Dr Andrea Berruti

National Research Council

Institute for Sustainable Plant Protection

viale Mattioli 25

Torino, 10125, Italy

andrea.berruti@unito.it

**Supplementary Table 2.** Total RNA quantity and quality as measured with the NanoDrop 1000 spectrophotometer (Isogen, The Netherlands) for each sample under study.

| Plant tissue | Weeks of treatment | Biological replicate | ng/μl | 260/280 | 260/230 |
| --- | --- | --- | --- | --- | --- |
| Leaf | 0 | A | 584 | 2.1 | 2.2 |
|  |  | B | 803 | 2.0 | 2.2 |
|  | 1 | A | 455 | 2.0 | 2.1 |
|  |  | B | 793 | 2.0 | 2.2 |
|  | 2 | A | 399 | 2.0 | 2.2 |
|  |  | B | 792 | 2.0 | 2.2 |
|  | 3 | A | 557 | 2.0 | 1.7 |
|  |  | B | 663 | 2.0 | 2.1 |
|  | 4 | A | 623 | 2.0 | 2.1 |
|  |  | B | 707 | 2.0 | 2.2 |
|  | 5 | A | 434 | 2.0 | 2.2 |
|  |  | B | 741 | 2.0 | 2.2 |
|  | 6 | A | 428 | 2.0 | 2.2 |
|  |  | B | 922 | 2.0 | 2.2 |
|  | 7 | A | 412 | 2.0 | 1.6 |
|  |  | B | 735 | 2.0 | 2.2 |
|  | 8 | A | 338 | 2.0 | 2.2 |
|  |  | B | 555 | 1.9 | 2.1 |
| Flower bud | 0 | A | 244 | 1.9 | 1.5 |
|  |  | B | 1380 | 1.9 | 2.2 |
|  | 2 | A | 152 | 1.9 | 1.9 |
|  |  | B | 1159 | 1.9 | 2.0 |
|  | 4 | A | 165 | 1.8 | 1.7 |
|  |  | B | 444 | 1.8 | 1.9 |
|  | 6 | A | 353 | 1.9 | 1.7 |
|  |  | B | 470 | 1.8 | 2.0 |
|  | 8 | A | 256 | 1.9 | 1.6 |
|  |  | B | 640 | 1.9 | 2.1 |
