# Supplementary material for: Cold Treatment Breaks Dormancy but Jeopardizes Flower Quality in Camellia japonica L
Source: Front Plant Sci. 2015 Nov 12;6:983. doi: 10.3389/fpls.2015.00983 (PMC4641915; doi:10.3389/fpls.2015.00983)
Supplement: Supplementary file 3 [file Table_3.DOCX]

***Supplementary Material***

**Cold treatment breaks dormancy but jeopardizes flower quality in *Camellia japonica* L.**

Berruti A.^1,2^, Christiaens A.^3^, De Keyser E. ^4^, Van Labeke M.C.^3^, Scariot V.^2^

**Corresponding author:**

Dr Andrea Berruti

National Research Council

Institute for Sustainable Plant Protection

viale Mattioli 25

Torino, 10125, Italy

andrea.berruti@unito.it

**Supplementary Table 3.** PCR efficiencies for each of the candidate genes under study. The standard error of the mean (SEM) calculated between two technical replicate standard curves is reported.

|  | PCR efficiency | SEM |
| --- | --- | --- |
| *CjARP* | 1.829 | 0.004 |
| *CjSuSy* | 1.879 | 0.01 |
| *CjCAP* | 1.977 | 0.012 |
| *CjDEH* | 1.989 | 0.03 |
| *CjERF* | 1.951 | 0.006 |
| *CjF3GalTase* | 1.816 | 0.006 |
| *CjANR* | 1.933 | 0.008 |
| *CjDFR* | 1.933 | 0.005 |
| *CjFLS* | 1.691 | 0.015 |
| *CjATPSb* | 1.853 | 0.004 |
| *CjNADH5* | 1.932 | 0.009 |
| *CjRNAPb* | 1.979 | 0.004 |
| *CjRS3P* | 1.760 | 0.009 |
