# Supplementary material for: Cold Treatment Breaks Dormancy but Jeopardizes Flower Quality in Camellia japonica L
Source: Front Plant Sci. 2015 Nov 12;6:983. doi: 10.3389/fpls.2015.00983 (PMC4641915; doi:10.3389/fpls.2015.00983)
Supplement: Supplementary file 4 [file Data_Sheet_1.DOCX]

***Supplementary Material***

**Cold treatment breaks dormancy but jeopardizes flower quality in *Camellia japonica* L.**

Berruti A.^1,2^, Christiaens A.^3^, De Keyser E. ^4^, Van Labeke M.C.^3^, Scariot V.^2^

**Corresponding author:**

Dr Andrea Berruti

National Research Council

Institute for Sustainable Plant Protection

viale Mattioli 25

Torino, 10125, Italy

andrea.berruti@unito.it

**Supplementary Data 1**. Candidate gene partial 5’-3’ sequences isolated from cDNA originated from RNA extracted from leaves of *Camellia* *japonica* ‘Nuccio’s Pearl’.

>CjF3GalTase

CGACATGGCAGAGGAGATGCGGGTCCCGTGGGTCCCACTTTGGAGTTCAGGGGCTTGCTCCTTGTCAACCCATTGCTACACTGACCTTATCAGGGAGACTGTTGGAATCCATGGCATTGCAGGACGCGAAAACGAAATCCTGAAATTCGTCCCAGGATT

>CjANR

TGTCCCTACTGATTTTGGAGATTTTCCATCGAAAGCGAAGTTGATCCTCTCGTCTGAGAAGCTTACCAAAGAGGGATTCAGTTTCA

>CjCAP

GGATCGGACAAATTGGAAGACCAACATTCTCACAACGCTTTTAGTTCCTTACATTTTCATGAGTCTACCTTCATTACTGTTTGGCTTTCTCAGGGGAGATATTGGAAAATGGGTCGCTTTCATTACTGTCATATTGCGCCTCTTCTTCCCTAAA

>CjDFR

AAGAGGCTGGTGTTCACATCCTCTGCTGGAACTGTTAATGTCCAGGAACACCAACAACCCATTTTCGACGAGAACAATTGGAGTGACTTGGATTTCATCAATAAGAAGAAGATGACTGGCTGGATGTATTTTGTTTCAAAAACATTGGCAGAGAAAGCAGCATGGGA

>CjDEH

GCCACACCACCACTAATAAATAAATACAATTTTCATGATATATCATATATGTAAATCGCTTGTTTGTGGCCAGAGCGCCAGAGTCTGCTAGTCTAGTTTATGATGTGTGTTTTGTGTGTTGTCATAAATGCGTT

>CjERF

TAAATGGGCTGCTGAGATCCGTGACCCAAGGAAAGGGGTCCGCGTTTGGCTTGGAACTTTCAACACAGCTGAAGAAGCTGCAAGAGCCTATGATGCCGAGGCAAGGAAGATTCGTGGCAAGAAAGCTAAGGT

>CjFLS

AGAATGAACAACCTGCAATCACCACAATCCAGGGCAAGGTCCTGGAAGTCCCGGTGATCGACCTCAGCAACGACTCGGACGACCAGAATATTGTCCAATTGGTCGCTGAGGCCTGCCGGGACTGGGGGATATTTCAGGTTGTAA

>CjSuSy

CCAAGGATTTGGAAGAGCAAGCCGAGATGAAGAAGATGTATGAGATGATTGAAACCTACAAGTTGCAGGGTCAATTTAGGTGGATTTCGTCACAGATGAACAGGGTGAGGAA

>CjARP

GTGTGGAGGAGCGTGTTCAACCCTGGTAGCAACCTTGCCACCAAGGGTATTGGTGCTGATGTCTTTGACAAGCCCCAGCCCAACTCTCCCGCTGTCTATGATTGGCTCTACAGTGGGGAGACGAGGAGC

>CjATPSb

AACCCACCCTTAGTACCGAAATGGGTACTTTACAAGAAAGAATTACTTCTACTAAAGAAGGGTCCATAACTTCTATTCAAGCAGTTTATGTACCTGCA

>CjRS3P

CCTTCAGAAAAACGAAGTTATTCAAGTTCTTTTTCCCAAAGAAGTCCCGCTCCGACGGACCGACGAGTCATCTACTTAAAAGGACCCTCC

>CjNADH5

AGCAAGGTGCTCCCCTTTATTTGAATACTCACCTACGGCTTTGATTGCTATTACTTTTGCAGGAGCTATGACGTCATTCCTTGCGGCAAC

>CjRNAPb

GCTACGAAAATCTTTGTCAATGGTTGCTGGGTTGGTATTCATCGTGATCCTGATATGTTGGTGAAGACACTAAGACGACTGAGGAGACGGGTA
